# Cattle Bile Aggravates Diclofenac Sodium-Induced Small Intestinal Injury in Mice

**DOI:** 10.1155/2011/315858

**Published:** 2011-04-11

**Authors:** Hironori Ishikawa, Shiro Watanabe

**Affiliations:** Division of Clinical Application, Institute of Natural Medicine, University of Toyama, 2630 Sugitani, Toyama 930-0194, Japan

## Abstract

Cattle bile (CB) has long been used in Japan as an ingredient of digestive medicines. Bile acids are major chemical constituents of CB, and CB ingestion is assumed to affect small intestinal injury induced by nonsteroidal anti-inflammatory drugs (NSAIDs). Mice were fed a diet supplemented with or without CB for 7 days and treated with diclofenac sodium (DIF) to induce small intestinal injury. Lesion formation was enhanced, and PGE2 content and COX expression levels were elevated in the small intestine of DIF-treated mice fed the CB diet compared with those fed the control diet. The administration of a reconstituted mixture of bile acids found in CB enhanced lesion formation in DIF-treated mice. CB administration elevated the contents of CB-derived bile acids in the small intestine, some of which exhibited a high cytotoxicity to cultured intestinal epithelial cells. These results suggest that the elevated levels of CB-derived cytotoxic bile acids in the small intestine contribute to the aggravation of DIF-induced small intestinal injury. The use of CB may be limited during the therapy of inflammatory diseases with NSAIDs.

## 1. Introduction

Nonsteroidal anti-inflammatory drugs (NSAIDs) are extensively used as antipyretics and analgesics. However, long-term ingestion of NSAIDs induces gastrointestinal side effects, such as lesion formation in the stomach and duodenum [1]. In addition, recent investigations have revealed that lesion formation and erosion in the small intestinal mucosa are induced more frequently than those in the stomach and duodenum [2]. Intestinal bleeding and anemia due to small intestinal injury are relevant in rheumatic patients taking NSAIDs [3–5]. Experimental studies have also confirmed that the administration of several types of NSAID in rats and mice can induce mucosal injury predominantly in the small intestine accompanied by intestinal inflammation and lesion formation associated with severe bleeding and blood loss [6, 7].

NSAIDs inhibit mucus secretion and increase the motility of the small intestine through the inhibition of prostaglandin synthesis by cyclooxygenase-1 (COX-1) [8]. These pathological responses facilitate bacterial translocation into the intestinal mucosa, which triggers various immunoinflammatory reactions, such as leukocyte infiltration and the generation of reactive oxygen species and proinflammatory cytokines [8]. Bacterial translocation also upregulates COX-2 expression, leading to the stimulation of prostaglandin synthesis in the small intestine [8]. However, this COX-2-dependent prostaglandin synthesis plays a protective role by attenuating the earlier pathological events due to the inhibition of COX-1-dependent prostaglandin synthesis by NSAIDs in the small intestine. However, NSAIDs also decrease COX-2 activity and thereby induce small intestinal injury. Thus, the decrease in both COX-1 and COX-2 activities is involved in the mechanism by which NSAIDs induce small intestinal injury. 

Bile acids associated with phospholipids in bile; therefore, their hydrophobicity and cytotoxicity for intestinal epithelia are attenuated [9]. However, it is shown that NSAIDs can liberate free bile acids from bile acid-phospholipid complexes. The liberated free bile acids are more potent in injuring intestinal epithelial cells than their complexes with phospholipids [10]. The ability of NSAIDs to liberate free bile acids from bile acid-phospholipid complexes is considered to be due to their ability to bind to phospholipids [11] or directly to bile acids [12]. In particular, the cytotoxicity of complexes of NSAIDs and bile acids is assumed to be extremely high [12]. Thus, the interaction of NSAIDs with phospholipids or bile acids can explain the mechanism by which NSAIDs induce small intestinal injury depending on the cytotoxicity of bile acids for intestine epithelial cells. There are several studies examining the effects of administration of bile acids on NSAID-induced small intestinal injury in the experimental animals. Oral administration of taurochenodeoxycholic acid ameliorated, but that of ursodeoxycholic acid exacerbated, small intestinal injury in indomethacin-treated rats [13]. In contrast, ursodeoxycholic acid could ameliorate ibuprofen-induced small intestine injury in rats [14]. Since taurochenodeoxycholate is a hydrophobic, but ursodeoxycholic acid is a relatively less hydrophobic bile acid, the effects of bile acids with different hydrophobicity on NSAID-induced small intestinal injury in the experimental animals were not simply reflected by physiochemical properties of bile acids.

Animal bile preparations harvested from different animal species, such as bear, cattle, and pig, have long been used, mainly in Asian countries. In particular, animal bile preparations are used as ingredients of digestive medicines in Japan. Bile acids are the major chemical constituents of animal bile preparations and facilitate the emulsification and hydrolysis of dietary fats by pancreatic lipases [15]. These properties explain the usefulness of animal bile preparations as an ingredient of digestive medicines. However, ingested bile acids are incorporated into the enterohepatic circulation and change the bile acid composition in bile and the small intestinal lumen [16, 17]. Therefore, the ingestion of hydrophobic bile acids might increase the hydrophobicity of the bile acids in bile or the small intestinal lumen. Among the animal bile preparations used in Japan, cattle bile (CB) is most extensively used as an ingredient of digestive medicines, but it is constituted of bile acids with a relatively high hydrophobicity, such as taurine and glycine conjugates of cholic and deoxycholic acids [15]. Thus, the ingestion of CB is suggested to elevate the levels of hydrophobic bile acids in bile and the small intestinal lumen. However, no study has exactly tested the effect of the administration of cattle bile as well as bile acids contained in this animal bile on NSAID-induced small intestinal injury. Therefore, it is necessary to clarify whether NSAID-induced small intestinal injury is aggravated by the administration of CB.

In this study, we examined the effect of CB administration on small intestinal injury induced by diclofenac sodium (DIF), the most extensively used NSAID in hospitals, in mice. Lesion formation, COX expression levels, and prostaglandin content in the small intestine were assessed as indices of small intestinal injury [8]. Furthermore, we investigated the role of bile acids in the modulation of DIF-induced small intestinal injury by CB administration by evaluating the changes in bile acid composition in the small intestine and the cytotoxicity of the bile acids found in the small intestine of mice for intestinal epithelial cells *in vitro*.

## 2. Materials and Methods

### 2.1. Animal Treatments

Male ddY mice (SLC, Shizuoka, Japan) at 5 weeks of age were acclimated in the environment of our animal room for 1 week before the treatments were started. CB used in this study was obtained as a semidry paste (Mitsuhoshi Pharmaceutical Co., Nara, Japan, Lot. A-007). Bile acid composition in CB was determined by high-performance liquid chromatography (HPLC; LC-10A, Shimadzu Co. Ltd., Kyoto, Japan) coupled with the use of an evaporative light-scattering detector (ELSD; Sedex 75, SEDERE, Alfortville, France) as described previously [18]; the composition is shown in Table 1. A powdered diet (CE-2, Clea, Tokyo, Japan) was used to prepare the control and CB-supplemented diets. CB was added to the basal diet at 0.2% (w/w), and mice were fed the test diet for 7 days. A nonsupplemented diet was used as the control diet. In the experiment to examine the effect of the reconstituted mixture of the bile acids found in CB, the basal diet was supplemented with a mixture of taurocholic acid (TCA), glycocholic acid (GCA), taurodeoxycholic acid (TDCA), and glycodeoxycholic acid (GDCA) at 39, 28, 3.4 and 9.8 mg/100 g diet, respectively, which were based on the proportions of these bile acids in CB (Table 1). These bile acids were purchased from Steraloids Inc. (Newport, RI). After the mice were fed the control and test diets for 7 days, DIF (Wako Pure Chem, Osaka, Japan) dissolved in distilled water was orally administered to the mice at 20 mg/kg body weight, and, thereafter, the same diets were administered for an additional 24 hours [19]. Mice fed the control diet were orally administered with only distilled water as the untreated control group. The protocols for this experiment were approved by the Committee for Animal Care and Experiments of the University of Toyama.

### 2.2. Assessment of Small Intestinal Lesion Formation

Mice were killed 24 hours after DIF administration by cervical dislocation under anesthesia with gaseous isoflurane (5% w/v in air), and the entire small intestine was harvested. Immediately, the small intestine was excised along antimesenteric lines and flattened on a dry filter paper. The small intestine was covered with another piece of filter paper and immersed into 10% formalin overnight to fix the tissues. The number and the sum of length of lesions in the small intestinal mucosa were determined under a stereoscope as described previously [19]. Measurement was performed by an observer who was blind to the treatments of sample specimens.

### 2.3. Determination of Contents of Bile Acids and Prostaglandin E2 in Small Intestine

Entire small intestines were harvested from the mice 24 hours after DIF administration and homogenized in 10 mL of 99.5% ethanol using Teflon potter homogenizers. The homogenates were centrifuged at 3000 rpm for 10 minutes, and the resultant supernatants were used for the determination of bile acid and prostaglandin E_2_ (PGE_2_) contents. Bile acids were extracted from an aliquot of ethanol extracts using octadecylsilyl-bonded silica cartridges (Sep-Pak C18, Waters, Milford, MA) as described previously [20]. Bile acid-containing fractions eluted from the cartridge were dried under nitrogen gas and dissolved in aliquots of the mobile phase used for HPLC. Details of the procedures for the determination of bile acids by HPLC were reported previously [16]. For the determination of PGE_2_ content, ethanol extracts from the small intestine prepared as described above were dried under nitrogen gas. PGE_2_ content in the samples was determined using enzyme immunoassay kits (Cayman Chemicals, Ann Arbor, MI) [21].

### 2.4. Western Blot Analysis of Cyclooxygenase Isoforms in Small Intestine

As described above, mice fed the control or CB diet for 7 days were treated with DIF or vehicle, and the small intestine was harvested. The small intestine was excised and washed in cold saline to remove luminal contents. The specimens were blotted onto dry filter papers to remove excess water. The expression levels of COX-1 and COX-2 in the small intestine were determined by western blot analysis as described previously [21].

### 2.5. Cytotoxicity of Bile Acids to Cultured Intestinal Epithelial Cells

The cytotoxicity of bile acids were compared using a cultured rat intestinal epithelial cell line, IEC-18 (DS Pharma Biomedical, Osaka, Japan) [22]. The cells growing in Dulbecco's minimum essential medium (MEM; Wako Pure Chem., Osaka, Japan) supplemented with 10% (v/v) fetal bovine serum and 5 *μ*g/mL insulin (Sigma-Aldrich, St. Louis, MO) under humidified air containing 5% CO_2_ were suspended in the same medium and treated with trypsin. The cells were divided into the wells of microplates at 2.5 × 10^4^ cells/well and incubated for 24 hours. Bile acids dissolved in the culture medium were added to the wells of microplates at final concentrations of 2 and 4 mg/mL in triplicate. The cells were further incubated for 24 hours and nonadhering cells were removed by washing with calcium- and magnesium-free phosphate-buffered saline (PBS) twice. Adhering cells were treated with 0.5% crystal violet (w/v) containing 20% methanol for 10 minutes. Excess dye was washed by immersing the plates in tap water. After the plates were dried, 1% (w/v) sodium dodecyl sulfate (SDS) was added (100 microL/well), and the absorbance at 595 nm was measured using a microplate reader (Multiscan FC, Thermo Scientific, Vantaa, Finland). Cell viability was expressed as the percentage of absorbance of the cells treated with bile acids with respect to that of the untreated cells.

### 2.6. Statistical Analysis

The statistical significance of the difference between the control- and CB-diet-fed groups was estimated by unpaired Student's *t*-test. For the analysis of the effects of DIF treatment and CB administration and their interaction, two-way analysis of variance (ANOVA) was used. Probability values below 0.05 are considered statistically significant.

## 3. Results

### 3.1. CB Aggravates DIF-Induced Lesion Formation in Small Intestine

**Figure 1:**
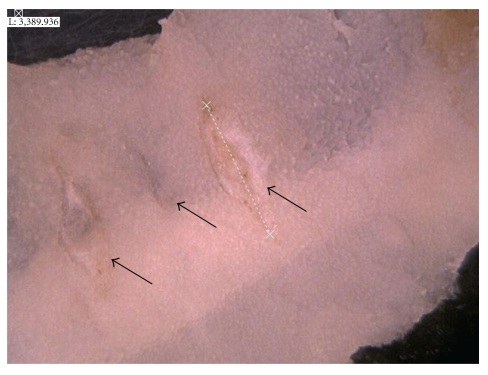
Typical image of lesioned small intestinal mucosa of DIF-treated mice. Arrows indicate the lesions formed along the line of mesenteric attachment of the small intestine. The dotted line indicates the length of the ulcers.

**Figure 2:**
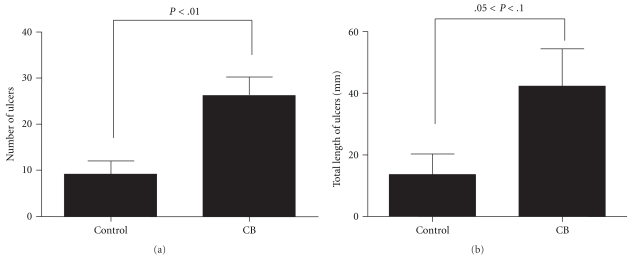
Effect of CB administration on the number (a) and length (b) of lesions formed in small intestine of DIF-treated mice. Mice were fed the control and CB diets, and lesion formation was estimated 24 hours after the oral administration of DIF. Columns and bars, respectively, represent the mean and SEM from six mice in each group. Statistical analysis was carried out by an unpaired Student's *t*-test.

A typical feature of small intestinal lesions of DIF-treated mice is shown in Figure 1. Lesions were formed along the lines of the mesenteric attachment of the small intestine of DIF-treated mice. The number of lesions formed in the small intestine of DIF-treated mice was significantly greater in the mice fed a diet supplemented with CB at 0.2% (w/w) than in the control group (*P* < .01; Figure 2). The total length of lesions tended to be greater in the CB group than in the control group (.05 < *P* < .1).

### 3.2. CB Increased COX Expression Levels and PGE_2_ Content in Small Intestine of DIF-Treated Mice

**Figure 3:**
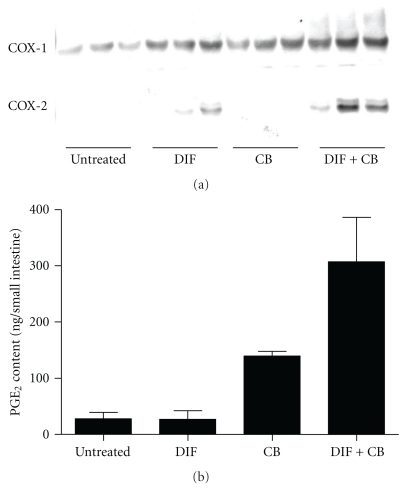
Effects of DIF treatment and CB administration on COX-1 and COX-2 expression levels and PGE_2_ content in small intestine. Mice fed the control or CB diet were treated with DIF or vehicle, and the expression levels of COX-1 and COX-2 (a) and PGE_2_ content (b) in the small intestine were determined by Western blot analysis and enzyme immunoassay, respectively. Three and 6 mice in each group were used for the determination of COX expression levels and PGE_2_ content in the small intestine, respectively. Fold difference in densitometric values of COX-1 bands compared with that of untreated group were 2.9, 3.0, and 5.7 in the CB-, DIF-, and CB/DIF-treated groups, respectively. PGE_2_ contents in the small intestine is expressed as mean + SEM. The probability values estimated in two-way analysis of variance were 0.0634, 0.0004, and 0.0634 for the effects of DIF treatment, CB administration, and their interaction, respectively.

COX-2 was undetectable in the small intestine from untreated mice, but was detectable in that from DIF-treated mice (Figure 3(a)). The administration of CB only did not induce the elevation of COX-2 in the small intestine of mice. However, combined treatments with CB and DIF markedly elevated the COX-2 expression level in the small intestine. In contrast, COX-1 was detectable even in the small intestine of untreated mice but its level tended to be elevated in the small intestine of mice treated with DIF or CB. In addition, the combined treatment with CB and DIF elevated the expression level of COX-1 in the small intestine. Although DIF treatment did not significantly change the PGE_2_ content in the small intestine, CB administration exerted significant effects on PGE_2_ content in the small intestine (two-way ANOVA, *P* < .001; Figure 3(b)). PGE_2_ content in the small intestine tended to be elevated by the interaction between CB and DIF treatments (two-way ANOVA, .05 < *P* < .1).

### 3.3. Reconstituted Mixture of CB-Derived Bile Acids Enhances Small Intestinal Lesion Formation Induced by DIF

**Figure 4:**
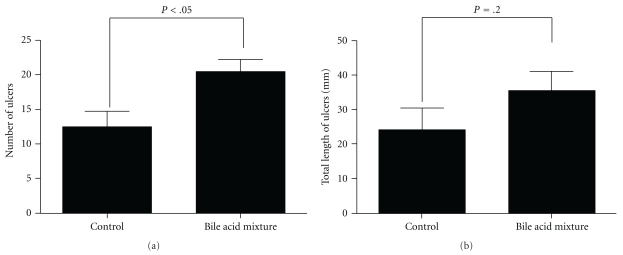
Effect of reconstituted mixture of bile acids contained in CB on number (a) and length (b) of lesions formed in small intestine of DIF-treated mice. Mice were fed the control diet and the diets supplemented with a mixture of bile acids contained in CB (Table 1), and lesion formation was estimated 24 hours after the oral administration of DIF. Columns and bars respectively represent the mean and SEM from six mice in each group. Statistical analysis was carried out by unpaired Student's *t*-test.

Mice were fed a diet supplemented with a reconstituted bile acid mixture composed of TCA, GCA, GDCA, and TDCA at concentration found in 0.2% CB diet for 7 days, and lesion formation in the small intestine of mice treated with DIF was compared with that of the mice fed the control diet (Figure 4). It was shown that the number of lesions in the small intestine of mice fed a diet supplemented with the bile acid mixture was significantly greater than that of mice fed the control diet (*P* < .05). The total length of lesions formed in the small intestine was slightly greater in the group fed the reconstituted bile acid mixture than in the control group, but this difference did not reach statistical significance (*P* = .20).

### 3.4. Bile Acid Composition in Small Intestine

**Figure 5:**
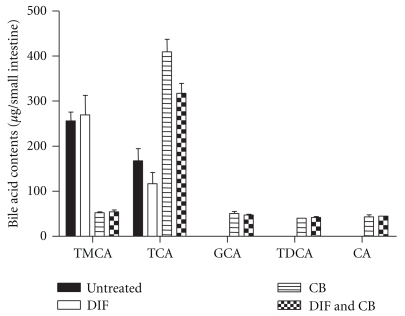
Effect of CB administration on contents of bile acids in small intestine. Mice fed the control or CB diet were treated with DIF or vehicle and the contents of bile acids in the small intestine were determined by HPLC. Columns and bars represent the mean and SEM of 3 to 6 mice in each group. Statistical analysis was carried out by two-way ANOVA. CB treatment significantly changed both TMCA and TCA contents in the small intestine (two-way ANOVA, *P* < .0001). DIF treatment significantly changed TCA content (*P* < .02) but not TMCA (*P* > .05) content in the small intestine. Interaction between DIF treatment and CB administration was not significant (*P* > .05) in terms of both TMCA and TCA contents in the small intestine.

The small intestine of untreated mice contained mainly tauromuricholic acid (TMCA) and TCA (Figure 5). In two-way ANOVA, CB administration (*P* < .001) but not DIF treatment (*P* > .05) significantly changed the contents of TMCA in the small intestine, although these treatments did not show significant interaction in changing TMCA content (*P* > .05). CB administration significantly elevated TCA content (*P* < .001) whereas DIF treatment reduced TCA content (*P* < .02) in the small intestine. However, the interaction between these treatments was not significant (*P* = .435). GCA and TDCA as well as CA were detectable in the small intestine of mice fed the CB diet, although the contents of these bile acids were not significantly changed by DIF treatment.

### 3.5. Cytotoxicity of Bile Acids to Cultured Intestinal Epithelial Cells

**Figure 6:**
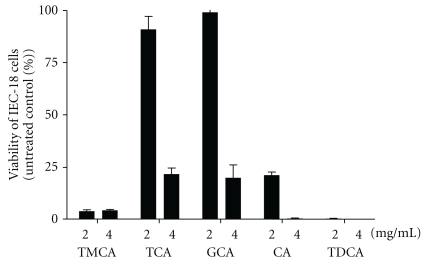
Cytotoxicity of bile acids for cultured intestinal epithelial IEC-18 cells *in vitro.* IEC-18 cells were treated for 24 hours with the bile acids found in the small intestine of mice administered with CB. The viability of treated cells was expressed as the percentage of absorbance of the cells treated with bile acids with respect to that of the untreated cells. Columns and bars represent the mean and SEM, respectively.

The intestinal epithelial cell line IEC-18 was used to estimate the cytotoxicity of bile acids found in the small intestine of mice fed the CB diet (Figure 6). Similar cytotoxicity was shown by TCA and GCA. The cytotoxicity of TMCA and TDCA were much greater than those of TCA and GCA, although CA exerted a moderate cytotoxicity.

## 4. Discussion

The COX-2 expression level was higher in the small intestine of mice treated only with DIF than in that of untreated mice (Figure 3(a)). In addition, the COX-1 expression level was slightly elevated in the small intestine of DIF-treated mice (Figure 3(a)). The elevation of COX-1 levels is supposed to be due to its expression in inflammatory cells or fibroblasts massively infiltrated into the inflamed small intestinal lesions 24 hours after the administration of DIF, (Figure 1). However, these responses were not associated with significant changes in PGE_2_ content in the small intestine (Figure 3(b)). These findings indicate that small intestinal injury induced by DIF is associated with inflammatory responses, although the upregulation of COX-1 and COX-2 expressions in the small intestine seems to be insufficient to elicit prostaglandin generation. In contrast, the small intestine of mice administered with only CB exhibited a significant increase in PGE_2_ content despite the absence of significant elevation of COX-2 expression level and only a small elevation of COX-1 expression level. These findings suggest that CB could upregulate PGE_2_ synthesizing ability mainly owing to the augmentation of availability of arachidonic acid for PGE_2_ synthesis. In addition, another possibility of the activation of PGE synthases might be due to the upregulation of PGE synthases (PGES) in the small intestine of mice administered with CB. Furthermore, it should be noted that the combined treatment with DIF and CB resulted in a robust increase in PGE_2_ content in the small intestine, which was accompanied by the elevation of COX-1 and COX-2 expression levels. These findings reflect the fact that immunoinflammatory reactions in the small intestine are enhanced as a result of the aggravation of small intestinal lesion formation through the synergism between DIF treatment and CB administration (Figure 2). Thus, PGE_2_ contents in the small intestine of DIF-treated mice was enhanced by CB administration, indicating that the aggravation of DIF-induced small intestinal injury by CB administration is not due, at the very least, to the inhibition of COX-mediated prostaglandin generation at 24 hours after DIF administration. However, there might be a case that CB administration could inhibit COX-mediated prostaglandin synthesis earlier than this time point. Effect of CB administration on the time-course changes in PGE_2_ contents during DIF-induced small intestinal injury should be further examined.

It can be concluded that bile acids contained in CB play a crucial role in the aggravation of small intestinal injury by CB administration in DIF-treated mice because the administration of a reconstituted mixture of bile acids found in CB also aggravated small intestinal injury in DIF-treated mice (Figure 4). However, the augmentation of lesion formation induced by the reconstituted mixture of bile acids was slightly lower than that induced by CB administration (Figure 2). This suggests that factor(s) other than bile acids could contribute, in part, to the aggravation of DIF-induced small intestinal injury. Upon CB administration, only a small but significant elevation of the contents GCA, TDCA, and CA, whose cytotoxicity to intestinal epithelial cells were extremely higher than those of endogenous bile acids in the small intestine (Figure 5), was observed. We, therefore, suggest that the incorporation of bile acids with higher hydrophobicity or cytotoxicity from CB into the small intestinal lumen is involved in the enhancement of small intestinal injury. On the other hand, the level of TMCA, the most abundant bile acid in the small intestine of mice, was reduced, but that of TCA was elevated in the mice fed the CB diet compared with those fed the control diet (Figure 5). Because TMCA is reported to be a much less hydrophobic bile acid [23], its cytotoxicity for IEC-18 cells was expected to be low. However, the cytotoxicity of TMCA was rather higher than those of bile acids with relatively high hydrophobicity, such as TCA, GCA, or CA (Figure 6). In addition, cytotoxicity of TMCA seemed to be similar to that of more hydrophobic TDCA, although more detailed comparison should be made for cytotoxicity of TMCA and TDCA in their lower concentration ranges. It has also been reported that the hydrophobicity of bile acids is not the only determinant of their cytotoxicity, as shown by *in vitro *tests [24]. There are possibilities that the cytotoxicity of TMCA depends on the types of cells or the culture conditions, such as concentration of serum. The role of the decrease in TMCA content in the small intestine in the enhancement of DIF-induced intestinal injury should be further investigated. However, cytotoxicity of bile acids for cultured intestinal epithelial cells was examined in the absence of DIF. It is known that bile acid cytotoxicity for epithelial cells can be augmented in the presence of NSAID [10]. Therefore, we should be more careful for the interpretation of cytotoxicity of different types of bile acids *in vitro* in considering the role of luminal bile acids in NSAID-induced small intestinal injury.

Interestingly, TCA was the only bile acid whose level in the small intestine was reduced in the DIF-treated groups (Figure 5), suggesting that TCA metabolism is selectively altered during DIF-induced small intestine injury. Changes in the rate of secretion of TCA into the intestinal lumen, deconjugation of TCA in the small intestine, or the absorption of TCA from the small intestine might be involved in the decrease in the level of TCA in the small intestine of DIF-treated mice. In addition, these factors determining the level of TCA in the small intestine might be directly affected by DIF. Effects of CB on the expression levels as well as the activities of transporters or metabolizing enzymes for bile acids in the small intestine or liver could elucidate the selective modulation of TCA level in the small intestine during DIF-induced small intestinal injury. In addition, we found that occurrence of unconjugated bile acid, cholic acid (CA) in the small intestines of mice administered with CB. This form of bile acid is suggested to be generated in the small intestinal lumen by deconjugation of TCA or GCA and can be assumed to play a role in the aggravation of small intestinal injury induced by DIF due to its high hydrophobicity. However, we have determined bile acid profiles in the small intestinal contents but not in bile. Therefore, it is difficult to predict whether the changes in TCA and CA levels in the small intestinal contents by the administration of CB and DIF (Figure 5) occurred in liver or small intestinal lumen. The determination of bile acid profiles in bile or liver would enable us to understand how CB or DIF administration induced the changes in the levels of bile acids in the small intestinal contents.

We cannot exclude the possibility that CB-derived bile acids enhance small intestinal injury independently of the changes in the cytotoxicity of bile acids in the small intestine. In fact, conflicting results have been reported on the effects of bile acids with different hydrophobicity on NSAID-induced small intestinal injury in the rats [13, 14]. It has already been shown that excretion of NSAIDs into bile is critical to the development of small intestinal injury; bile duct ligation can attenuate the secretion of NSAIDs into bile, which is associated with the attenuation of small intestinal injury in experimental animals [25, 26]. In addition, the ingestion of CB might change the absorption, distribution, metabolism, and elimination of DIF. Bile acids affect the expressions of metabolizing enzymes and transporters responsible for the behavior of DIF [27]. We can suppose that forms of bile acids (conjugated versus unconjugated) or the types of NSAIDs (indomethacin versus ibuprofen) might be related to the above conflicting results. The different chemical forms of bile acids might exert differential interaction with different types of NSAIDs in their enterohepatic circulation systems. Since DIF is known to undergo enterohepatic circulation [28], it is also possible that DIF interacts with bile acids in the metabolic processes during this system. We have already confirmed that the levels of blood DIF following its oral administration were not modified in the mice fed the CB diet compared with those fed the control diet (data not shown). However, we have not determined the levels of metabolites of DIF in blood and in enterohepatic circulation. In particular, the levels of DIF and its metabolites, such as glucuronide, are important in determining the extent of small intestinal injury [29]. Further detailed studies are necessary to elucidate the effect of CB on small intestinal damage from the viewpoint of the levels of DIF and its metabolites. 

In Japan, the range of dosages of CB used as an ingredient in regular digestive medicines is from 50 to 100 mg/day. However, the maximal allowance of daily intake of CB as an ingredient of digestive medicines is 500 mg, which contains approximately 170 mg of cholate and 33 mg of deoxycholate (Table 1). Epidemiological studies would be helpful to estimate the harmfulness of intake of CB and NSAID in combination, although no such study has been carried out. However, a study on humans [16] has shown that the ingestion of cholate at 15 mg/kg body weight/day, approximately 900 mg/day when the average body weight is assumed to be 60 kg, for 14 days results in approximately two-fold higher levels of cholate in the aspirates from the small intestine than those measured before the treatment. This change is close to that induced in the small intestine of mice fed the diet supplemented with 0.2% CB (Figure 6). Therefore, ingestion of cholate at a regular dosage (50 to 100 mg/day) as well as CB even at the maximum dosage (500 mg/day) would cause only smaller changes in the intestinal bile acid composition than the above-mentioned condition reported by Woollett et al. [16]. Instead, cholate dosage as in the above-mentioned human study may cause harmful effects when taken in combination with NSAIDs. Since only a single dosage of CB (0.2%) was tested for DIF-induced small intestinal injury in the present study, it is still unclear to what extent of aggravation of DIF-induced small intestinal injury could be induced by the administration of lower dosages of CB. An approach is also required to clarify the correlation between the changes in bile acid composition in the small intestinal lumen and severity of small intestinal injury in the experimental animals. We should further investigate to elucidate the mechanism by which cattle bile aggravates DIF-induced small intestinal injury from viewpoints of the modification of hydrophobicity of bile acids in the small intestines and of metabolic interaction between bile acids and DIF in enterohepatic circulation systems.

## Figures and Tables

**Table 1:** Bile acid composition of cattle bile.

Bile acids	Proportion (%, w/w)
Taurocholic acid (TCA)	19.5
Glycocholic acid (GCA)	14.0
Glycodeoxycholic acid (GDCA)	1.7
Taurodeoxycholic acid (TDCA)	4.9
